# Exploring sex-based differences in patient outcomes: A secondary analysis of Heartwatch, an Irish cardiovascular secondary prevention programme^☆^

**DOI:** 10.1016/j.ijcrp.2025.200376

**Published:** 2025-02-14

**Authors:** Ivana Keenan, Fintan Stanley, Robyn Homeniuk, Joseph Gallagher, Michael O'Callaghan, Claire Collins

**Affiliations:** a Irish College of GPs, 4-5, Lincoln Pl, Dublin, D02 XR68, Ireland; b ALONE, Olympic House, Pleasants Street, Dublin 8, D08 H67X, Ireland

**Keywords:** Sex differences, Risk factors, Secondary prevention, General practice

## Abstract

**Background:**

In the last two decades, sex-related differences regarding cardiovascular diagnosis, treatment, and risk factors management have been reported. The current study aims to explore differences in cardiovascular outcomes among male and female patients attending the Irish secondary cardiovascular prevention programme - Heartwatch.

**Methods:**

This is a retrospective observational study. Anonymous data was extracted from the Heartwatch database from 2003 to 2017. Cardiovascular risk factors were analysed at sign-up and at four years follow-ups. An 8-point aggregate risk score (CCare Score) was assessed to calculate targeted outcomes. Generalized estimating equations models were applied for data analysis.

**Results:**

In total 8893 patients (77 % male) were included. Females exhibited a higher risk profile across all cardiovascular risk factors and were more likely to be off target than males at baseline and after 4 years of programme attendance [M to F odds ratios(95 % CI); systolic blood pressure: 1.35 (1.21–1.49), waist circumference: 2.11(1.89–2.36), physical activity: 1.72 (1.53–1.95)]. CCare scores also demonstrated the gap between male and female patients at baseline [mean(sd); M: 5.1(1.2), F: 4.8(1.2)] and after 4 years of structured care [mean(sd); M: 5.3(1.2), F: 4.9(1.2)]. Female patients were less likely to be prescribed aspirin and ACE inhibitors but more likely to be prescribed AT2 inhibitors, calcium channel blockers, and diuretics compared to male patients.

**Conclusions:**

The Heartwatch programme has demonstrably improved patient care, however, the continuous underperformance of female patients necessitates further investigation to ensure appropriate and equitable secondary CVD prevention among the Irish population.

## Highlights

• Sex-based differences in CVD risk outcomes were identified in secondary CVD prevention programme.
• Females had worse CVD risk factors at baseline, and after 4 years of care.
• Females are less likely to receive aspirin/ACE inhibitors, but more likely to get AT2 inhibitors/diuretics than males.
• Further research needed to understand underlying causes and ensure optimal care for both sexes.

## 1 Introduction

Cardiovascular disease (CVD) is one of the leading causes of morbidity and mortality worldwide [1,2]. Within the European Union (EU), CVD negatively impacts the lives of more than 60 million people, accounting for one-third of all deaths and 20 % of premature deaths (before the age of 65) [3].

Previous studies highlighted sex-related differences in prevalence and treatment for CVD, and control of CVD risk factors [[4], [5], [6], [7]]. The research suggested that although males have higher CVD incidents and hospital admission rates; mortality rates, as well as poorer prognosis following an acute CVD event, are higher in females [[4], [5], [6], [7]]. The results from the series of EUROASPIRE studies also indicated that compared with males, females have worse CVD risk factor profiles and are less likely to adhere to lifestyle changes and achieve preventative targets [[8], [9], [10]]. According to these studies, female patients are more likely to suffer from higher cholesterol levels, diabetes, obesity, and have less physical activity, than male patients, whereas male patients were more likely to be smokers [8,9].

Long-term alterations of lifestyle significantly reduce the risk of CVD [11,12], and therefore preventative programmes have been developed to target modifiable CVD risk factors among at-risk populations. Secondary CVD prevention programmes play a key role in promoting behaviour change in patients with established diseases to improve their health outcomes and prevent future cardiovascular events. Considering the historically misleading conception that females have a lower risk of CVD than males, female patients were typically less targeted by primary and secondary CVD prevention programmes [13,14], and therefore less likely to be assigned to adequate programmes, and receive evidence-based medical therapy [14,15,16].

Since 2003, the national programme for secondary prevention of CVD – “Heartwatch” – has taken place in Irish general practice [17,18]. The Heartwatch programme represents a strategic approach to reducing morbidity and mortality caused by CVD and initially was based on the second European Task Force Guideline [17,18]. The previous evaluations of the Heartwatch programme highlighted that over time significant improvements in blood pressure control, LDL cholesterol, and smoking were noted, however little or no improvements on average were shown in BMI, waist circumference, and physical activity [[17], [18], [19], [20]]. The most recent study on Heartwatch published in 2023, identified that the patients’ sex was predictive of better scores; where male patients who attended the Heartwatch programme were more likely to achieve better overall results in risk factors management, compared with female patients [19]. Building on the results from the previous study, the present paper aims to explore sex-based differences in CVD outcomes in greater depth, with a particular focus on attendance patterns, risk factor management, and pharmacological treatment. Obtaining a more comprehensive understanding of the mechanisms behind sex-based differences in CVD outcomes would aid the effectiveness of future prevention programmes for both sexes.

## 2 Methods

### 2.1 Heartwatch programme overview

Heartwatch is a national, structured, cardiovascular secondary prevention programme in Ireland led by general practice [17]. The programme was developed using international secondary prevention guidelines [21,22] to create a standard protocol for the continuing care of patients with a history of cardiac events. In total, 20% of all general practitioners (GPs) in Ireland (n = 480) were selected to participate in Heartwatch [17].

The programme aimed to reduce morbidity and mortality by monitoring CVD risk factors and treatments. Patients with a history of acute myocardial infarction (AMI), as well as coronary interventions, including Coronary Artery Bypass Graft (CABG), or Percutaneous Transluminal Coronary Angioplasty (PTCA), were eligible for the programme [17]. In addition, although both private and public patients could have enrolled in the Heartwatch programme, the majority of patients had public status [17] and received care under the GMS Scheme and therefore accessed their GP consultations at no cost. Once admitted to the programme, they attended an initial consultation with their GP, followed by up to four visits a year. At each visit, the patient and their general practice team reviewed physical and medical measurements and provided brief interventions on diet, physical activity, and smoking cessation. Heartwatch was designed to capture a change within individual patients, based on their attendance in the programme [17].

### 2.2 Setting and inclusion criteria

The present study is a retrospective observational study. Data items analysed include consultations from January 2003 to March 2017. On average, patients have been attending the Heartwatch programme for 8 years, however, within the 8 years of attendance, the majority of the consultations took place within 4 years of signup. The inclusion criteria for the study were: i) patients had to have a history of AMI, CABG, or PTCA; and ii) patients had to have at least one visit per year for four consecutive years. If patients had more than one qualifying event (QE), counts and intervals were calculated based on their earliest recorded QE occurrence.

Some of the patients who attended a structured diabetes care programme, without QE, also attended the Heartwatch programme, however, due to differing treatment approaches they were excluded from the analysis.

### 2.3 Outcome measures and targets

The recommendations [21,22] applied for the Heartwatch programme proposed the targets regarding the patients’ lifestyle choices (physical exercise level >210min/week of moderate exercise; smoking cessation; and healthy food choices) as well as physical, laboratory and therapeutic measurements (systolic BP < 140 mmHg; HbA1c < 53 mmol/mol; waist circumference female <80 cm, male <94 cm; and prescribed an anti-coagulant or anti-platelet agent; and prescribed a lipid-lowering agent). Only the target level for lipoprotein cholesterol (LDLc) was updated from 3.0 mmol/L to 1.8 mmol/L^22^, and these changes were applied to the Heartwatch programme.

Following EUROASPIRE studies [8,9,10] and the methods used by Ergatoudes et al. [23], a summary measure to assess overall adherence to recommended targets was developed. A summary measure named a Continuing Care (CCare) score was developed to count all individuals' risk factor outcomes. Since the dietary information was not available, the CCare score was based on seven CVD risk factors including ‘Blood pressure’, ‘Cholesterol levels’, ‘Glucose levels’, ‘Waist circumference’, ‘Physical activity’, ‘Smoking’ and ‘Pharmacological treatment’ (Table 1). The patients would gain a point (score) for each outcome that was within the target. The exception was Diabetes Mellitus (DM), where patients who were not diagnosed with DM would score two points, due to the high prevalence of comorbidity of CVD and DM [24]. A patient could gain a maximum score of 8, across seven outcomes.

**Table 1:**
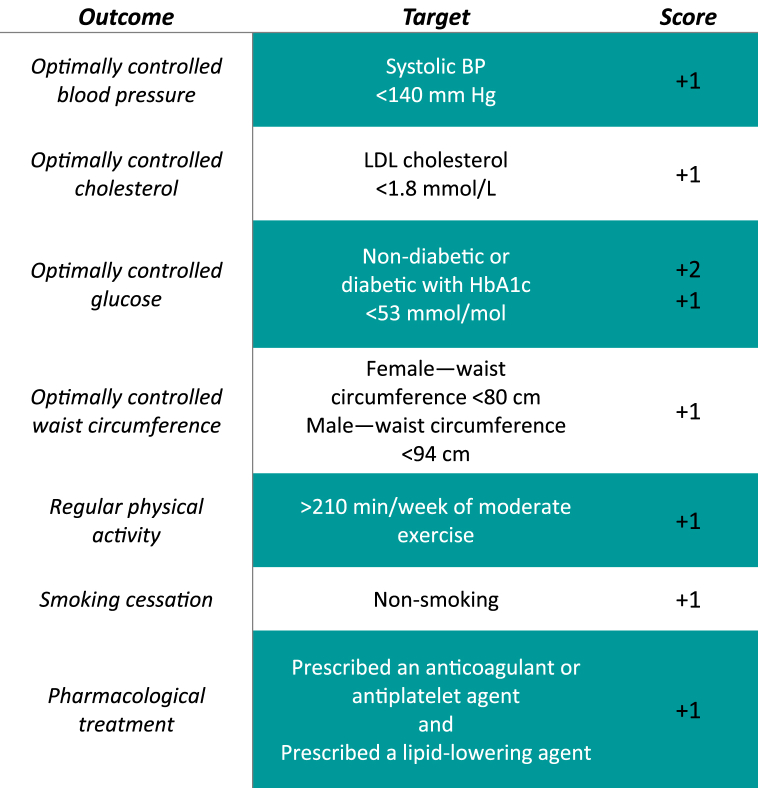
Components, target levels, and scoring used to calculate the Continuing Care (CCare) score outcome measure.

∗ BP, blood pressure; HbA1c, hemoglobin A1C; LDL, low-density lipoprotein.

The development of the CCare score was scrutinised, and rectified by the research team, consisting of GP specialists in cardiology and diabetes, researchers, and data experts.

### 2.4 Statistical analysis

Cohort demographics and programme interactions are summarized using descriptive statistics. Health measures and prescriptions are reported by means and proportions. Sex-based inequalities are reported as male-female odds ratios with 95 % CI.

To account for repeated measures and multiple variables, generalized estimating equations (GEE) models were applied to assess the relationship between cardiovascular risk, patient attributes, and behaviours. The models were trained on a 10 % data subset, with the final model including seven significant parameters: sex, age at signup, year of signup, qualifying event type, qualifying event interval, years in HW, and the interval between HW visits.

Analysis was performed using IBM SPSS Statistics 27 and R (V.4.1) with RStudio (V.1.4).

## 3 Results

The analysis of data from the cohort revealed notable sex-based differences across multiple domains, including baseline demographics, health metrics, and pharmacological treatments. The following sections outline the key findings, highlighting areas where divergences between male and female patients were observed over the initial four-year period of programme enrolment.

### 3.1 Cohort demographics & patterns of care

The enrolment period for this study spanned from 2003 to 2017.

**Fig. 1:**
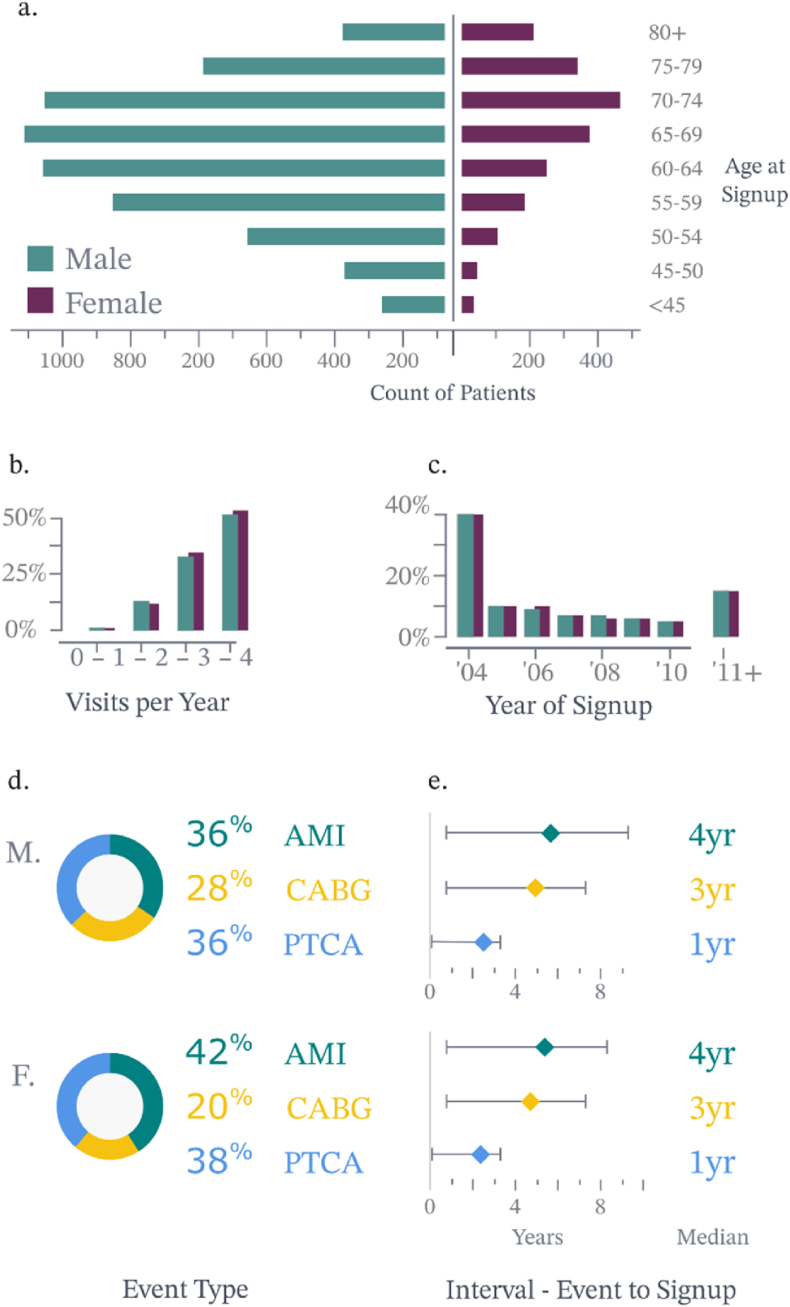
Heartwatch overview 2003–2017. (A) Population pyramid of all patients grouped by age and sex. (B) All Heartwatch visits grouped by year of visit. (C) All Heartwatch visits grouped by visits per year. (D) All patients grouped by earliest qualifying event type. (E) All patients grouped by interval from the earliest qualifying event to the date of the first Heartwatch visit.

The cohort comprised 8893 patients, and the majority of patients in the study were male (77 %) and tended to enrol in the Heartwatch programme at younger ages than female patients (the male median age at sign-up was 65 compared with 70 for females). The age distribution of the cohort ranged broadly from 27 to 101 years, with a median age of 66 years (Fig. 1a).

The median year of signup was 2004, with no significant difference observed between male and female patients (Fig. 1b). The visit frequency to the programme was also investigated where the median number of visits per year was three, with 60 % of patients attending three or four times annually. This pattern was consistent across both sexes (Fig. 1c).

In terms of patients’ QE, 42 % of females had an AMI, 20 % had a CABG and 38 % had PTCA. Comparatively, 36 % of males had an AMI, 28 % had a CABG, and 36 % had a PTCA (Fig. 1d).

Another aspect of the study was the interval between the qualifying event and programme signup, which ranged from 0 to 20 years. The median interval was 2 years for the entire cohort, with no difference between the sexes. However, the mean interval differed slightly, being 3.9 years for female patients and 4.2 years for male patients (Fig. 1e).

### 3.2 Health measures

**Fig. 2:**
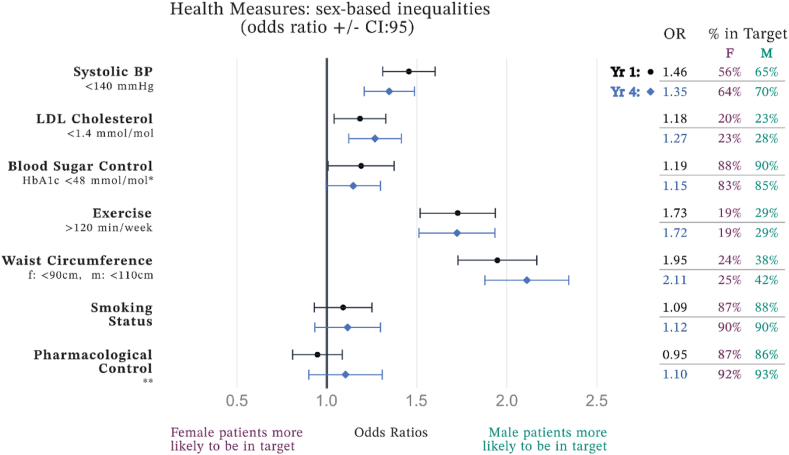
Sex differences in risk factor management. ∗ Blood Glucose Control is otherwise scored 0,1,2 as defined in Table 1, here non-diabetic scored as 1 (instead of 2) to allow OR calculations. ∗∗ Pharmacological control definition is available in Table 1.

Systolic blood pressure showed improvement in both sexes over the four years, with females improving from a mean of 136 mmHg–134 mmHg and males from 133 mmHg to 131 mmHg. The odds of male patients being within the target range were higher initially (OR = 1.46, 95 % CI: 1.32–1.61) and remained higher after four years (OR = 1.35, 95 % CI: 1.21–1.49) (Fig. 2).

In terms of LDL Cholesterol, both female and male patients demonstrated significant improvement. Female patients reduced their mean LDL level from 2.53 mmol/mol to 2.28 mmol/mol, while male patients reduced from 2.42 mmol/mol to 2.20 mmol/mol. Males had higher odds of achieving the target LDL level both at the start (OR = 1.18, 95 % CI: 1.05–1.34) and after four years (OR = 1.27, 95 % CI: 1.13–1.42) (Fig. 2).

The analysis of weekly moderate exercise revealed a pronounced sex-based difference. Initially, female patients reported an average of 179 min per week, increasing to 187 min by year four. Male patients started at 231 min, improving to 237 min. The odds ratios highlighted a significant difference, with males having higher odds of meeting the exercise target at the outset (OR = 1.95, 95 % CI: 1.74–2.18) and an even higher likelihood after four years (OR = 2.11, 95 % CI: 1.89–2.36) (Fig. 2).

### 3.3 CCare scores

**Fig. 3:**
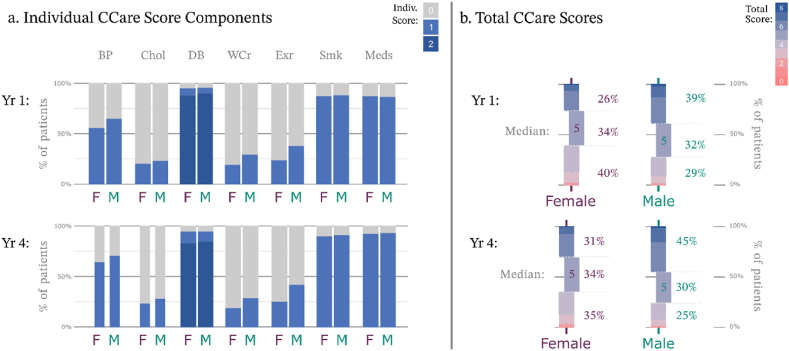
Comparison of CCare scoring by sex (a) Comparison of total CCare scores by percentages of cohort, median values highlighted with % at, above, and below median indicated. (b) Comparison of each of the 7 components of the CCare score.

In line with the sex-based differences in the mean values of key risk factors, male patients were more likely to be on target for almost all of the modifiable risk factors monitored in Heartwatch. For SBP, waist circumference, and physical activity targets, the proportion of female patients in target after four years never reached the proportion of males in target after one year (Fig. 3a). At the end of the first year in Heartwatch, 53 % of female patients reached target levels of SBP, which increased to 55 % by year four, whereas 62 % of male patients had SBP on target after year one and 67 % after year four (Fig. 3a). A similar pattern of sex-based differences was observed in physical exercise. After the first year, 38 % of males reached the target level, increasing by 42 % by the fourth year. In contrast, 24 % of females met the target level after the first year, with a slight increase to 25 % by the fourth year (Fig. 3a).

The comparison of total CCare scores over the 4-year period revealed that males achieved more targets than females during the programme. After one year, 71.9 % of males had achieved a five or higher CCare score compared to just 60.1 % of females. Less than a third of females reached five or higher after four years, indicating they never reached the level male patients attained after one year (Fig. 3b).

### 3.4 Pharmacological treatments

**Fig. 4:**
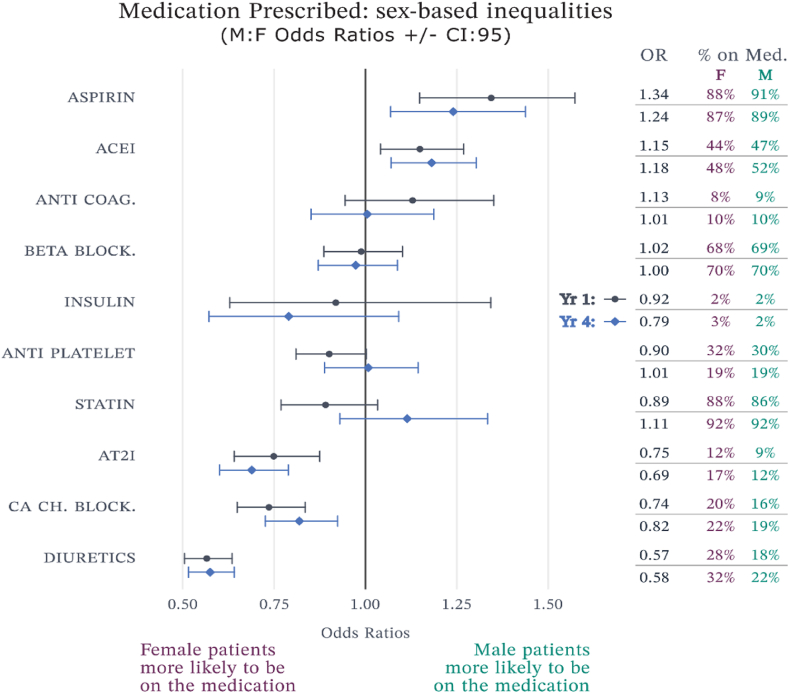
Sex differences in medication prescribing.

In the first year, a sex-based disparity in aspirin prescription was observed, with 88 % of female patients and 91 % of male patients receiving the medication (OR = 1.34, 95 % CI: 1.15–1.56). This statistically significant difference indicates a 34 % higher odds of aspirin prescription for male patients compared to female patients. By the fourth year, while the overall prescription rates slightly decreased to 87 % in females and 89 % in males, the disparity narrowed (OR = 1.24, 95 % CI: 1.07–1.44), indicating a narrowing disparity (Fig. 4).

A sex-based disparity in angiotensin-converting-enzyme inhibitors (ACEi) prescription was observed. In the first year, 44 % of female patients and 47 % of male patients received ACEi (OR = 1.15, 95 % CI: 1.04–1.27). By the fourth year, usage increased to 48 % in females and 52 % in males, with the disparity persisting (OR = 1.18, 95 % CI: 1.07–1.30) (Fig. 4).

A sex-based difference was noted in calcium channel blocker prescriptions. In the first year, 20 % of female patients and 18 % of male patients received these medications (OR = 0.74, 95 % CI: 0.65–0.84). By the fourth year, usage increased to 22 % in females and 19 % in males (OR = 0.82, 95 % CI: 0.73–0.92) showing a consistent selection for calcium channel blockers among females (Fig. 4).

A significant sex-based disparity was observed in diuretic prescriptions. Initially, 28 % of female patients and 18 % of male patients received diuretics (OR = 0.57, 95 % CI: 0.51–0.64). By the fourth year, usage increased to 32 % in females and 22 % in males, with the disparity persisting (OR = 0.58, 95 % CI: 0.52–0.64) (Fig. 4).

## 4 Discussion

### 4.1 Summary

The analysis of data from the cohort revealed notable sex-based differences across multiple domains, including baseline demographics, health metrics, and pharmacological treatments over the initial four-year period of the Heartwatch programme enrolment.

The majority of patients in the study were male (77 %) and tended to enrol in the Heartwatch programme at younger ages than female patients. Prior to enrolment to Heartwatch, 42 % of females had a history of AMI, in comparison with 36 % of men. Despite a higher proportion of female patients being enrolled within a year of their qualifying event they typically started with worse CVD risk factor values across the board.

While both sexes showed improvements in multiple health metrics over the four years, male patients consistently had higher odds of achieving target health measures, particularly in systolic blood pressure, LDL cholesterol, waist circumference, and weekly exercise.

Investigation of the overall performance, conducted through an 8-point CCare score displayed male patients achieving a greater number of targets than females during the programme. After one year, 39 % of males had achieved a five or higher CCare score compared to just 26 % of females. Although CCare scores improved for both sexes in year four (45 % of males reached a five or higher CCare score compared to 31 % of females) - female patients’ improvements during their Heartwatch participation did not reach the same level of improvements as male patients, even after four years of receiving the same standardised care. Male patients consistently performed better.

The analysis also highlighted significant sex-based differences in the prescription of key cardiovascular medications. Female patients were generally less likely to be prescribed aspirin and ACE inhibitors but more likely to be prescribed AT2 inhibitors, calcium channel blockers, and diuretics compared to male patients. These differences could be due to variations in clinical presentations, physician prescribing practices, or patient responses and tolerances.

Overall, our study demonstrated that sex-based differences in CVD risk factors in patient outcomes prevail and that females are more likely to have worse CVD risk factor profiles; and less likely to achieve desirable targets. Therefore, further investigations are needed, and future research studies should take into consideration patients' and GPs' perspectives and experiences with secondary CVD prevention programmes. The exploration of patients' attitudes (including knowledge of CVD risk factors, benefits, and barriers to engagement with CVD secondary prevention programmes), communication patterns (patients' communication preferences and GPs communication strategies), and potential GP biases (assessment of GPs’ understanding of sex differences in CVD risk factors, and treatment recommendations) from a qualitative perspective may contribute to a better understanding of the underlying causes and ensuring both female and male patients receive optimal, evidence-based care.

### 4.2 Comparison with existing literature and implications in the practice

Historically, females were largely underrepresented in CVD trials, resulting in CVD risk among females remaining underrecognized and undertreated for decades [25]. Timely identification and management of lifestyle risk factors through primary and secondary prevention programmes stand as paramount for the improvement of overall cardiovascular health. Although significant steps took place to target both sexes, inequalities in the assessment of the risk factors and subsequent CVD prevention between male and female patients remained [25,26].

Most of the previous research highlighted that female patients were less likely to achieve targeted outcomes for cholesterol and glucose levels, as well as for physical activity and non-obesity [[7], [8], [9], [10],^25^]. The INTERHEART trial, which was conducted across 52 countries, found that females with higher SBP and elevated cholesterol levels are at greater risk of AMI than males [27], and in keeping with these findings, the Swedeheart study found a lower target achievement in blood pressure control and lipid control among female patients [28]. Females were also more prone to less physical activity and a sedentary lifestyle than men [25]. Consistent with previous research, our analysis confirmed significant, persistent sex-based differences in health outcomes, suggesting key roles for sex-specific biological or behavioural factors. The rationale behind the sex-based gap within CVD morbidity and mortality is frequently attributed to differences in cardiovascular physiology and pathophysiology between females and males. These distinctions originate from reproductive hormone differences (hormonal changes during menopause, pregnancy, polycystic ovary disorders) as well as the development of autoimmune diseases, which affect females disproportionally higher than males [25,26]. Nevertheless, a range of under-recognized risk factors, encompassing psychosocial elements such as depression and anxiety, abuse and intimate partner violence, and health illiteracy, are closely associated with elevated incidence and prevalence of CVD among females [25,26]. Recognizing these factors is crucial in understanding and addressing the widening gap in CVD outcomes.

In line with previous research [29,30] our study revealed that in comparison with male patients, females were less likely to be prescribed aspirin and ACEi, and more likely to be prescribed AT2i, CaCHBlock, and Diuretics, at the start of the treatment. The previous research identified that the difference in prescribing patterns is due to a combination of doctor and patient-related factors; as doctor-related factors or under-prescription is often based on the preconceived perception that females are at lower risk of severe outcomes from CVD than men, and patient-related factors are based that females often hold more negative perceptions of medicine intake and are less likely to adhere to medication prescribed [[31], [32], [33], [34]]. Some of the previous studies also found that females were less likely to be prescribed statins in comparison with men [31,32], however, our study did not arrive at the same conclusion, as there was no notable sex-based difference or shift in the 4 years of attendance to the programme.

Although the perception that females have lower CVD risk has been disproven, many healthcare providers still tend to underestimate the cardiovascular risk for females, as well as sex-based differences. One study which assessed 47,841 patients, found that after being diagnosed with CVD, female patients were 20%–50 % less likely to receive information and guidance-directed therapies on future steps and prevention [12]. Overall, female patients are found to be less likely to enrol and adhere to cardiac rehabilitation programmes than male [35,36,37]. Our study findings that 77 % of patients who participated in the Heartwatch programme were male, reflects similar trends seen in rehabilitation programmes internationally. For instance, 2019 national reports from the UK and Australia highlighted that approximately 70 % of attendees in cardiac rehabilitation programmes were male, with only 30 % female [[38], [39]], aligning with findings from other studies [[40], [41], [42]]. A lower level of enrolment of the female cohort in cardiovascular prevention programmes is often attributed to a range of factors, as females are more likely to have negative perceptions towards the programmes, have less social support to join the programmes, and also experience various psychosocial barriers [35,43]. Considering that females are more often subjected to health disparities in comparison with men, further initiatives targeting specifically female patients are needed. A prerequisite for addressing disparities lies in the establishment of tailored and culturally sensitive guidelines, aiming to facilitate the improvement of knowledge among medical practitioners and female patients, in fostering a more equitable provision of CVD care. Females also have sex-specific risk factors such as gestational diabetes and pre-eclampsia which are associated with cardiovascular disease in later life. Sex-specific guidelines incorporating risk factor assessment, CVD prevention, and treatments focused on females, are urgently needed in Ireland, to ensure equitable healthcare and improvement of overall health and wellbeing outcomes for females.

As our study provides evidence that the Heartwatch programme was applied more readily to male patients, we must strive to ensure future chronic disease initiatives, such as the current chronic disease management (CDM), do not repeat this disparity in care delivery. From the Health Service Executive's (HSE) first report on the CDM programme, it seems this currently active programme is more successful at registering female patients than Heartwatch programme [46]. Female patients made up 62 % of asthma cases and 50 % of COPD cases. However, males tended to represent a larger proportion of IHD (63 % male), Atrial fibrillation (59 % male), and Diabetes mellitus cases (58 % male). Thus, it seems that the CDM programme - a chronic disease management programme as opposed to a secondary prevention initiative - appears to be more balanced in terms of gender representation than the Heartwatch programme, with a greater proportion of female participants. By identifying and managing chronic conditions earlier in both men and women, the CDM programme has the potential to significantly reduce future health risks and improve long-term outcomes across the population. However, given our findings, continued examination of sex differences is important for the CDM programme and general practice-delivered care. Education initiatives on chronic condition management should focus on promoting equitable representation across all groups, ensuring that everyone can benefit from high-quality primary care to help them manage their chronic disease(s).

### 4.3 Study limitations

Considering the size of the cohort (accounting for 8893 patients), and the length of time the observation took place (over four years), the study provided a rich overview of patient characteristics and outcomes. The analysed information was derived from patients’ medical records and therefore contributes to better accuracy of the collected information and mitigates potential biases associated with selection and recall.

Regarding limitations, the study was not a randomized control trial, and therefore there are no comparative or control groups, which may undermine internal validity.

Secondly, patients who were selected in the study attended the Heartwatch programme more regularly, on average 3.5 times a year. Across the entire Heartwatch database, many of the patients attended the programme fewer times a year, or for shorter periods. Therefore, the patients selected in this study, may not have been a representative sample, as by selecting patients who attended the programme 2-, 4- years of follow-up visits the results may have overestimated real changes assuming that withdrawals had less favourable results.

Finally, it is important to acknowledge the changes in CVD guidelines and treatments during our study period. The Heartwatch programme adhered to European guidelines on CVD prevention [21,22], which evolved over the years, resulting in some of the recommendations within various guideline publications being altered (e.g. LDL cholesterol was updated from 3.0 mmol/L to 1.8 mmol/L). Therefore, there is a possibility that patients may have received different treatments based on the recommendations provided in particular time periods. It is also important to note that although clinical guidelines updates on SBP did not occur during the study period, the European Society of Cardiology produced new guidelines on the management of blood pressure in 2021, and 2024 [44,45]. The 2024 guideline introduced a category of 'Elevated Blood Pressure', where the SBP target ranges between 120 and 129 mmHg for most patients receiving medication for lowering blood pressure [45]. Therefore, future research studies should consider aligning research objectives with new guidance, to include a comparative element that reflects both, old and new SBP targets to evaluate the benefits of secondary prevention programmes. Finally, since the start of the Heartwatch programme, pharmacological management of cardiovascular disease has been altered by new evidence and advice on medications such as aspirin, direct oral anticoagulants, beta-blocker types, and newer agents that act on the renin-angiotensin-aldosterone system. However, while shifts in medication prescribed in the Heartwatch programme reflect the increased emphasis on evidence-based patient-centered care for secondary CVD prevention, it was beyond the scope of this study to examine specifically how the introduction of different pharmacotherapeutic guidelines may have influenced these shifts and how such shifts may have altered patient outcomes.

## 5 Conclusions

A CVD secondary prevention programme delivered in Irish general practice has demonstrably improved patient care.

However, a clear propensity for GPs to “think secondary CVD prevention” more readily for male patients is apparent, with far more male patients being invited by GPs to participate in the programme.

In addition, male patients seem to respond better to the programme across a number of metrics, which may be a function of GP factors, patient factors and/or other unknown factors. In any case, more involvement of female patients in the design of programmes like Heartwatch seems to be warranted based on our findings.

## CRediT authorship contribution statement

**Ivana Keenan:** Writing – review & editing, Writing – original draft, Methodology, Conceptualization. **Fintan Stanley:** Writing – review & editing, Visualization, Methodology, Formal analysis, Data curation, Conceptualization. **Robyn Homeniuk:** Writing – review & editing, Conceptualization. **Joseph Gallagher:** Writing – review & editing, Conceptualization. **Michael O'Callaghan:** Writing – review & editing, Conceptualization. **Claire Collins:** Writing – review & editing, Supervision, Conceptualization.

## Acknowledgement of grant support

No additional funding was obtained to undertake this secondary analysis. The Irish 10.13039/100018270Health Service Executive funds the Heartwatch programme.

## Declaration of competing interest

Any potential conflicts of interest, including related consultancies, shareholdings and funding grants:
