# Supplementary material for: Exploring sex-based differences in patient outcomes: A secondary analysis of Heartwatch, an Irish cardiovascular secondary prevention programme
Source: Int J Cardiol Cardiovasc Risk Prev. 2025 Feb 14;24:200376. doi: 10.1016/j.ijcrp.2025.200376 (PMC11910684; doi:10.1016/j.ijcrp.2025.200376)
Supplement: Multimedia component 1 [file mmc1.docx]

# Supplementary Statistical Methods

## 1. Data Prep and Model Development

#### 1.1 Sample Selection

- Used complete case analysis for patients with 4 years of follow-up data
- Training-validation split (90:10) stratified by sex
- Time variables standardized:
  - Visit intervals converted to months
  - Follow-up time converted to years
  - Age maintained in years

#### 1.2 Model Specification

Four competing models were evaluated:

1. Base model with main effects
2. Model including lagged CCare scores
3. Model with sex-age interaction
4. Complex model with multiple interactions

All models were fitted using Generalized Estimating Equations (GEE) with:

- Gaussian family distribution
- Identity link function
- Various correlation structures tested (indep, AR1)
- Patient ID as clustering variable

#### 1.3 Model Selection Criteria

Models were compared using:

- QIC (Quasi-likelihood Information Criterion)
- RMSE (Root Mean Square Error) on validation set
- MAE (Mean Absolute Error) on validation set
- Clinical interpretability

## 2. Model Diagnostics

#### 2.1 Residual Analysis

- Residual plots against fitted values
- Partial residual plots for each predictor by sex
- Autocorrelation analysis of residuals

#### 2.2 Sensitivity Analyses

- Comparison of adjusted vs unadjusted odds ratios
- Testing of different correlation structures
- Assessment of model stability across patient subgroups
- Use of a lagged variable for previous outcomes
- More complex models including splines and non-linear terms were evaluated but did not improve model performance or alter key findings.

##

## 3. Model Summary

To analyse the relationship between sex and cardiovascular outcomes while accounting for repeated measures over time, we employed Generalized Estimating Equations (GEE). After systematic comparison of multiple model specifications, a model with independence correlation structure and patient-level clustering emerged as optimal based on the Quasi-Information Criterion (QIC).

The final model included sex, qualifying event type, qualifying event interval, and visit frequency as significant predictors. While age at signup and year of signup were tested, they did not significantly contribute to the model. Model validation on a held-out test set (10% of data) demonstrated good predictive performance with an RMSE of 1.18 and MAE of 0.94.

The model coefficients highlighted the persistent significance of sex in determining health outcomes (Wald statistic = 235, p < 2e-16), even after accounting for other factors. This robust statistical framework allowed us to evaluate the role of sex and other factors in influencing longitudinal health outcomes while appropriately handling the temporal dependencies in the data.

## Model Details

Table X: Results from Generalized Estimating Equation Model of CCare Scores

| Variable | Estimate | Std. Error | Wald Statistic | P-value |
| --- | --- | --- | --- | --- |
| Sex (M vs F) | 0.392 | 0.026 | 235.39 | <0.001 |
| PTCA | 0.128 | 0.026 | 23.34 | <0.001 |
| CABG | 0.045 | 0.028 | 2.52 | 0.112 |
| Time in Programme (years) | 0.036 | 0.003 | 109.92 | <0.001 |
| Year of Entry | 0.014 | 0.003 | 20.91 | <0.001 |
| QE Interval (months) | -0.002 | 0.0002 | 58.16 | <0.001 |
| Age (years) | -0.006 | 0.001 | 25.96 | <0.001 |
| Visit Interval (months) | -0.042 | 0.007 | 33.82 | <0.001 |

Notes:

- Model used independence correlation structure
- Estimated scale parameter: 1.4 (SE: 0.016)
- Analysis based on 8,646 clusters with maximum cluster size of 20
- Dependent variable: CCare Score
